# Computational analysis of ventricular mechanics in hypertrophic cardiomyopathy patients

**DOI:** 10.1038/s41598-023-28037-w

**Published:** 2023-01-18

**Authors:** Joy Mojumder, Lei Fan, Thuy Nguyen, Kenneth S. Campbell, Jonathan F. Wenk, Julius M. Guccione, Theodore Abraham, Lik Chuan Lee

**Affiliations:** 1 Department of Mechanical Engineering, Michigan State University, East Lansing, MI USA; 2 Department of Cardiology, University of California San Francisco, San Francisco, CA USA; 3 Department of Physiology & Division of Cardiovascular Medicine, University of Kentucky, Lexington, KY USA; 4 Department of Mechanical Engineering, University of Kentucky, Lexington, KY USA; 5 Department of Surgery, University of California San Francisco, San Francisco, CA USA

**Keywords:** Biomedical engineering, Cardiac hypertrophy

## Abstract

Hypertrophic cardiomyopathy (HCM) is a genetic heart disease that is associated with many pathological features, such as a reduction in global longitudinal strain (GLS), myofiber disarray and hypertrophy. The effects of these features on left ventricle (LV) function are, however, not clear in two phenotypes of HCM, namely, obstructive and non-obstructive. To address this issue, we developed patient-specific computational models of the LV using clinical measurements from 2 female HCM patients and a control subject. Left ventricular mechanics was described using an active stress formulation and myofiber disarray was described using a structural tensor in the constitutive models. Unloaded LV configuration for each subject was first determined from their respective end-diastole LV geometries segmented from the cardiac magnetic resonance images, and an empirical single-beat estimation of the end-diastolic pressure volume relationship. The LV was then connected to a closed-loop circulatory model and calibrated using the clinically measured LV pressure and volume waveforms, peak GLS and blood pressure. Without consideration of myofiber disarray, peak myofiber tension was found to be lowest in the obstructive HCM subject (60 kPa), followed by the non-obstructive subject (242 kPa) and the control subject (375 kPa). With increasing myofiber disarray, we found that peak tension has to increase in the HCM models to match the clinical measurements. In the obstructive HCM patient, however, peak tension was still depressed (cf. normal subject) at the largest degree of myofiber disarray found in the clinic. The computational modeling workflow proposed here can be used in future studies with more HCM patient data.

## Introduction

Hypertrophic cardiomyopathy (HCM) is a genetic heart disease that is associated with sudden cardiac death. This disease has a prevalence of less than 1 per 500 and a mortality rate that is four-fold higher in young adults than the general US population^1–6^. Most treatments (e.g., septal myectomy and pharmacological treatments) are designed to alleviate symptoms and decrease the risk of sudden cardiac death^7^ and recently, the treatment of HCM patients with a drug mavacamtem has produced substantial improvement in the cardiac function^8^. Hypertrophic cardiomyopathy can be classified generally into two phenotypes based on whether the left ventricular outflow tract (LVOT) is obstructed (obstructive HCM) or not (non-obstructive HCM)^9,10^. In both phenotypes, myofiber disarray is a histopathological hallmark^11^ that is either confined to some particular region in the left ventricle (LV) or is distributed throughout the entire LV. Besides myofiber disarray, HCM is also associated with other key histopathological features such as asymmetrical septal hypertrophy in the LV, changes in the myocardial contractility, and cardiac fibrosis^12–20^. In obstructive HCM patients, a pressure gradient $> 50\,\, \mathrm{mmHg}$ across the LVOT, either at resting or provoked condition, is also present^21,22^. These features are associated with changes in the LV function seen in HCM patients, such as a reduction in (global and segmental) longitudinal and circumferential strains^6,23–25^, a reduction in maximal force generating capacity^26–28^, an increase in relative adenosine triphosphate consumption during tension generation^29^, and a reduction in myocardial work (pressure-strain loop area)^30^.

Given the multiple histopathological features present in HCM patients, how each of these features contributes to the changes in the LV function is not clear. Although clinical studies can help reveal abnormalities of myocardial structure (e.g., myofiber disarray) associated with HCM^31^, the causal link of these features to LV function is difficult to ascertain in these studies. As such, the relative contribution of these remodeling features (i.e., asymmetrical hypertrophy, myofiber disarray) to the impairment of LV function in HCM patients still remains unclear. Mathematical modeling can help resolve this issue by quantifying the causal effects of the remodeling features to changes in the LV function in HCM patients. In relation to HCM, a few computational models have been developed to investigate the effects of remodeling features on LV function^32,33^. Specifically, mathematical models based on an idealized ellipsoidal LV geometry have been developed to investigate how regional strain is affected by myofiber disarray^34^ and sarcomeric mutation^32^. A computational study has been conducted to quantify the effects of remodeling features associated with HCM by perturbing the heart geometry of a healthy volunteer that is used as a baseline^33^. These studies, however, do not consider the difference in LV mechanics between obstructive and non-obstructive HCM. They also did not consider patient-specific LV geometries that encapsulate the heterogeneous distribution of wall thickness associated with this disease. Other computational studies are focused only on obstructive HCM^35,36^, but they did not consider the effects of myofiber disarray.

To address these limitations, we developed patient-specific finite element (FE) LV models based on clinical measurements acquired from patients with 2 different types of HCM (obstructive and non-obstructive) and a control subject. These models were constructed based on patient-specific LV geometries that were segmented from cardiac magnetic resonance images of these subjects. The models were coupled to a closed-loop circulatory model and calibrated using patient-specific clinical measurements of the LV volume waveform, blood pressures and peak global longitudinal strain (GLS). Contractile function of the cardiac muscle fibers in the 3 subjects was determined by the calibration. The calibrated models were then applied to investigate the effects of different degrees of myofiber disarray on LV function in both the obstructive and non-obstructive HCM subjects.

## Methods

### Clinical data

**Figure 1:**
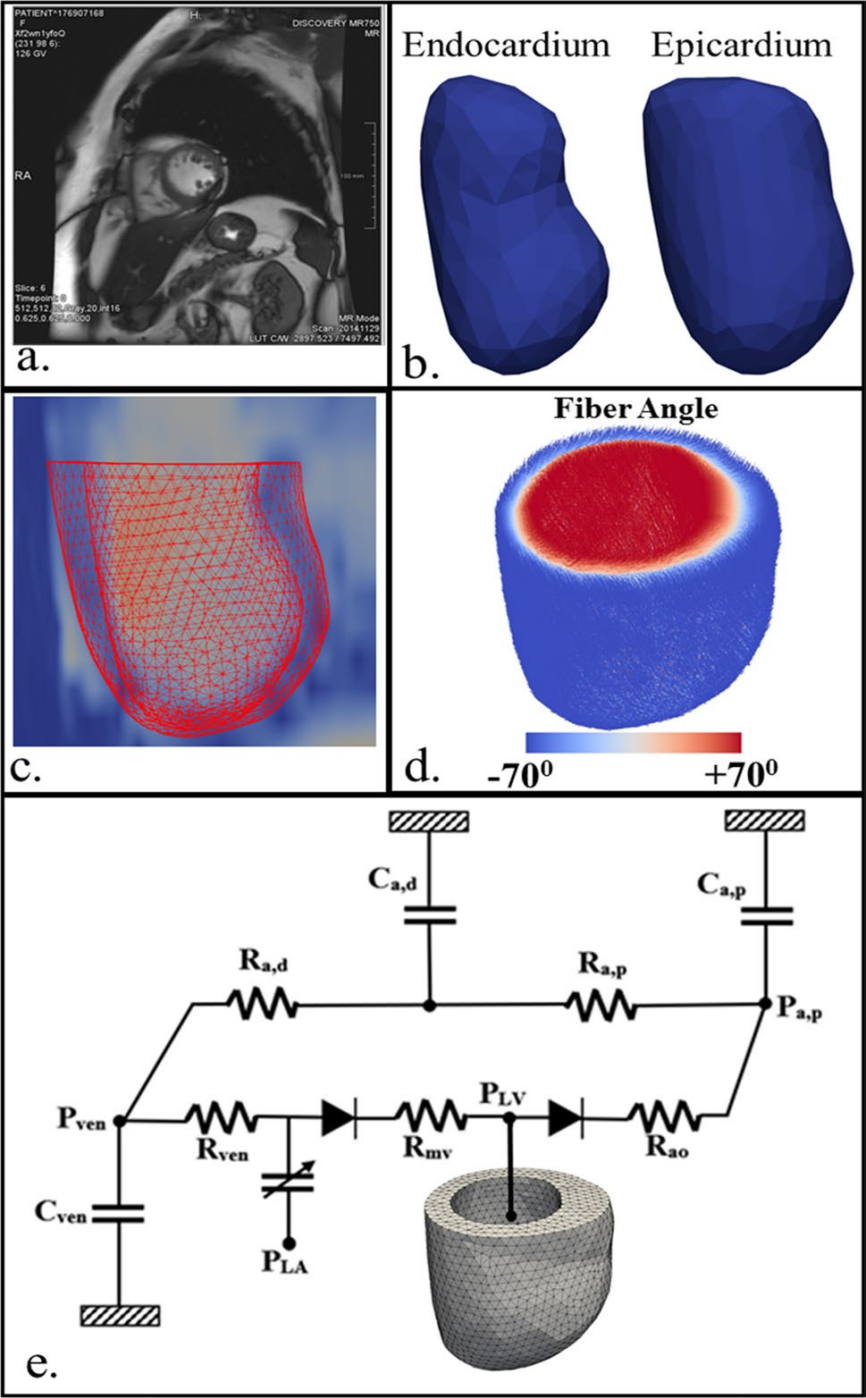
Construction of the LV FE model. (**a**) MR image segmentation; (**b)** segmented endocardium and epicardium of the LV; (**c**) FE model overlaid on the MR image in a long axis view; (**d)** transmural variation of mean myofiber direction across the LV wall; (**e**) schematic representation of LV FE model coupled with a closed-loop circulatory model. A sample representation is shown for the non-obstructive HCM patient.

Clinical data of 2 female HCM patients (obstructive and non-obstructive) along with a control female subject were acquired from the University of California San Francisco Medical Center (UCSFMC). This study was conducted in accordance with the principles of the Declaration of Helsinki and met the requirement of medical ethics. The UCSF Institutional Review Board of UCSFMC approved this research. As our study was purely retrospective in nature and used anonymized data, patient approval and informed consent were waived by the UCSF Institutional Review Board of UCSFMC. The data consists of cardiac magnetic resonance (MR) images, blood pressure measurements and peak global longitudinal strain (GLS) estimated from 3D echocardiographic images. Left ventricular (LV) cavity volume waveform of each subject was estimated by segmenting the endocardial wall in the MR images (Fig. 1a) over the cardiac cycle using MeVisLab (MeVis Medical Solutions AG). The clinical data are listed in Table 1.

**Table 1:** Clinical measurements of each subject.

Parameters	Control	Obstructive	Non-obstructive
Age (years)	69	57	61
Weight (kg)	58.1	97	75
Heart rate (bpm)	60	51	66
End diastolic volume (ml)	63	114	82
End systolic volume (ml)	18	38	12
Ejection fraction (%)	70	66.8	85.3
Global longitudinal strain (%)	− 20	− 13	− 19
Body surface area (m^2^)	1.56	2.04	1.72
Blood pressure (mm Hg)	126/65	151/80	133/66

### Reconstruction of LV FE model

Left ventricular endocardial and epicardial surfaces were segmented from the MR images associated with end-diastole (Fig. 1b). Patient-specific 3D LV geometries were then reconstructed from these surfaces and a FE mesh was generated for each geometry. The meshes consist of approximately 13,000 tetrahedral elements (Fig. 1c). Mean myofiber direction ${{\varvec{e}}}_{{{\varvec{f}}}_{0}}$ (Fig. 1d) was prescribed based on a linear transmural variation of the helix angle from + 70° at the endocardium to − 70° at the epicardium across the LV wall using a Laplace-Dirichlet rule-based algorithm^37^.

### Computational framework

The computational framework consists of the LV FE model, left atrium (LA), the proximal (a,p) and distal (a,d) arterial and venous (ven) compartments that are connected in a closed-loop circulatory system (Fig. 1e)^38–40^. In the framework, the rate of change of volume in each compartment of the circulatory system is described by the difference between the inflow and outflow rates of the connecting vessels i.e.,

1a $$\frac{{dV_{{LA}} \left( t \right)}}{{ dt}} = q_{{ven}} \left( t \right) - q_{{mv}} \left( t \right),$$

1b $$\frac{d{V}_{LV}\left(t\right)}{dt}={q}_{mv}\left(t\right)-{q}_{ao}\left(t\right),$$

1c $$\frac{d{V}_{a,p}\left(t\right)}{dt}={q}_{ao}\left(t\right)-{q}_{a,p}\left(t\right),$$

1d $$\frac{d{V}_{a,d}\left(t\right)}{dt}={q}_{a,p}\left(t\right)-{q}_{a,d}\left(t\right),$$

1e $$\frac{d{V}_{ven}\left(t\right)}{dt}={q}_{a,d}\left(t\right)-{q}_{ven}\left(t\right).$$

Flow rate associated with each compartment of the circulatory system depends on the prescribed compartment’s resistance (${R}_{mv}$, ${R}_{ao}$,${R}_{a,p}$, ${R}_{a,d}$,${R}_{ven}$) and the pressure difference across the compartment (i.e., pressure gradient). The flow rates are given as

2a $${q}_{ao}\left(t\right)=\left\{\begin{array}{c}\frac{{P}_{LV}\left(t\right)-{P}_{a,p}\left(t\right)}{{R}_{ao}}; if{P}_{LV}>{P}_{a,p}\\ 0; if{P}_{LV}<{P}_{a,p}\end{array},\right.$$

2b $${q}_{a,p}\left(t\right)=\frac{{P}_{a,p}\left(t\right)-{P}_{a,d}\left(t\right)}{{R}_{a,p}},$$

2c $${q}_{a,d}\left(t\right)=\frac{{P}_{a,d}\left(t\right)-{P}_{ven}\left(t\right)}{{R}_{a,d}},$$

2d $${q}_{ven}\left(t\right)=\frac{{P}_{ven}\left(t\right)-{P}_{LA}\left(t\right)}{{R}_{ven}},$$

2e $${q}_{mv}\left(t\right)=\left\{\begin{array}{c}\frac{{P}_{LA}\left(t\right)-{P}_{LV}\left(t\right)}{{R}_{mv}}; if{P}_{LA}>{P}_{LV}\\ 0; if{P}_{LA}<{P}_{LV}\end{array}\right.,$$

where ${R}_{ao}$ and ${R}_{mv}$ are the resistances of aortic and mitral valve, respectively.

Contraction of the LA is described using a time varying elastance function $e\left(t\right)$^41–43^. Specifically, pressure in the LA ${P}_{LA}\left(t\right)$ is prescribed to be a function of the volume ${V}_{LA}\left(t\right)$ by the following equations

3a $${P}_{LA}\left(t\right)=e\left(t\right){P}_{es,LA}\left({V}_{LA}\left(t\right)\right)+\left(1-e\left(t\right)\right){P}_{ed,LA}\left({V}_{LA}\left(t\right)\right),$$

3b $${P}_{es,LA}\left({V}_{LA}\left(t\right)\right)={E}_{es,LA}\left({V}_{LA}\left(t\right)-{V}_{0,LA}\right),$$

3c $${P}_{ed,LA}\left({V}_{LA}\left(t\right)\right)={A}_{LA}\left({e}^{{B}_{LA}\left({V}_{LA}\left(t\right)-{V}_{0,LA}\right)}-1\right),$$

3d $$e\left(t\right)=\left\{\begin{array}{c}\frac{1}{2}\left(sin\left[\left(\frac{\pi }{{t}_{max}}\right)t-\frac{\pi }{2}\right]+1\right);0<t\le \frac{3}{2}{t}_{max}\\ \frac{1}{2}{e}^{-\left(t-\frac{3}{2}{ t}_{max}\right)/\tau }; t>\frac{3}{2}{t}_{max}\end{array}\right..$$

In Eqs. (3a–3d), ${E}_{es,LA}$ is the end-systolic elastance of the atria, ${V}_{0,LA}$ is the volume-intercept of the end-systolic pressure volume relationship, and both ${A}_{LA}$ and ${B}_{LA}$ are parameters of the end-diastolic pressure volume relationship (EDPVR) of the atria. In the elastance function $e\left(t\right)$ given in Eq. (3d), ${t}_{max}$ and $\tau$ are the time taken to reach maximal chamber elastance and the relaxation time constant, respectively.

On the other hand, pressure in each (arterial and venous) compartment depends on its prescribed compliance and the difference between the instantaneous volume and its prescribed resting volume by

4a $${P}_{ven}\left(t\right)=\frac{{V}_{ven}\left(t\right)-{V}_{ven,0}}{{C}_{ven}},$$

4b $${P}_{a,p}\left(t\right)=\frac{{V}_{a,p}\left(t\right)-{V}_{a,p,0}}{{C}_{a,p}},$$

4c $${P}_{a,d}\left(t\right)=\frac{{V}_{a,d}\left(t\right)-{V}_{a,d,0}}{{C}_{a,d}},$$

4d $${P}_{LV}\left(t\right)= {f}^{FE}\left({V}_{LV}\left(t\right)\right),$$

where $${V}_{ven,0}, {V}_{a,p,0}, {V}_{a,d,0}$$ are constants representing the resting volumes and $${C}_{ven}, {C}_{a,p}, {C}_{a,d}$$ are total compliances of the venous, proximal and distal arteries, respectively. Finally, pressure in the LV, $${P}_{LV}\left(t\right)$$, is calculated from the FE model as described in the next section with the instantaneous volume, $${V}_{LV}\left(t\right)$$, as the input. The ODEs in Eqs. (1a–1e) are solved using an explicit time integration scheme. At each time step $${t}_{i}$$, the ventricular pressure, $${P}_{LV,i}$$ is computed from the FE model (Eq. 4d) based on the Lagrangian functional in Eq. (5) with the prescribed volume $${V}_{LV,i}$$ determined from the ODE (Eq. 1b). The computed ventricular pressure is then used to calculate the flow rates $${q}_{ao,i}$$ and $${q}_{mv,i}$$ using Eqs. (2a) and (2e), respectively. These flow rates are then used to calculate the ventricular volume at the next time step $${V}_{LV,i+1}$$ using Eq. (1b). We note here that during the isovolumic phases, which occur when $${P}_{LV}<{P}_{a,p}$$ in Eq. (2a) and $${P}_{LA}<{P}_{LV}$$ in Eq. (2e), $${V}_{LV}$$ remains unchanged from the previous time step (i.e., $${V}_{LV,i+1}$$=$${V}_{LV,i})$$ and $${P}_{LV}$$ is computed based on Eq. (5) as in the other non-isovolumic phases.

### Finite element model formulation

Finite element formulation of the LV model has been described previously^38–44,46^. Briefly, denoting *z* as the apex-to-base axis and *x*, *y* are axes orthogonal to *z*, the functional relationship between pressure and volume of the LV in Eq. (4d) is obtained based on the Lagrangian functional given by,

5 $$\mathcal{L}\left({\varvec{u}},p,{P}_{LV},{c}_{x},{c}_{y},{c}_{z} \right)={\int }_{{\Omega }_{0}}W\left({\varvec{u}}\right)dV- {\int }_{{\Omega }_{0}}p\left(J-1\right)dV-{ P}_{LV}\left({V}_{LV}\left({\varvec{u}}\right)-{V}_{LV}\right)-{c}_{x}\cdot {\int }_{{\Omega }_{0}}{u}_{x} dV-{\boldsymbol{ }\boldsymbol{ }\boldsymbol{ }\boldsymbol{ }\boldsymbol{ }\boldsymbol{ }\boldsymbol{ }\boldsymbol{ }\boldsymbol{ }\boldsymbol{ }\boldsymbol{ }\boldsymbol{ }\boldsymbol{ }\boldsymbol{ }\boldsymbol{ }\boldsymbol{ }\boldsymbol{ }\boldsymbol{ }\boldsymbol{ }\boldsymbol{ }\boldsymbol{ }\boldsymbol{ }\boldsymbol{ }\boldsymbol{ }\boldsymbol{ }c}_{y}\cdot {\int }_{{\Omega }_{0}}{u}_{y} dV-{c}_{z}\cdot {\int }_{{\Omega }_{0}}{\varvec{z}}\times {\varvec{u}} dV.$$

In Eq. (5), ${\varvec{u}}$ is the displacement field, $p$ is a Lagrange multiplier to enforce incompressibility of the tissue (i.e., Jacobian of the deformation gradient tensor $, J=1$), ${P}_{LV}$ is the Lagrange multiplier to constrain the LV cavity volume ${V}_{LV,\mathrm{cav}}\left({\varvec{u}}\right)$ to a prescribed value ${V}_{LV}$^47^. Both ${c}_{x}$ and ${c}_{y}$ are Lagrange multipliers to constrain rigid body translation in *x*, *y* directions and ${c}_{z}$ is the Lagrange multiplier to constrain rigid body rotation^48^. The functional relationship between the cavity volumes of the LV to the displacement field is given by,

6 $${V}_{LV}\left({\varvec{u}}\right)= \underset{{\Omega }_{k,inner}}{\int }dv=-\frac{1}{3}\underset{{\Gamma }_{k,inner}}{\int }{\varvec{x}}.{\varvec{n}} da ,$$

where ${\Omega }_{k,inner}$ is the volume enclosed by the inner surface ${\Gamma }_{k,inner}$ and the basal surface at $z = 0$, and ${\varvec{n}}$ is the outward unit normal vector.

The first variation of the Lagrangian functional in Eq. (5) leads to the following expression:

7 $$\delta \mathcal{L}\left({\varvec{u}},p,{P}_{LV},{c}_{x},{c}_{y},{c}_{z}\right)={\int }_{{\Omega }_{0}}\left({\varvec{P}}-p{{\varvec{F}}}^{-T}\right):\nabla \delta {\varvec{u}} dV- {\int }_{{\Omega }_{0}}\delta p\left(J-1\right)dV-{P}_{LV,\mathrm{cav}}{\int }_{{\Omega }_{0}}cof\left({\varvec{F}}\right):\nabla \delta {\varvec{u}} dV - \delta {P}_{LV}\left({V}_{LV}\left({\varvec{u}}\right)-{V}_{LV}\right)-{\delta c}_{x}\cdot {\int }_{{\Omega }_{0}}{u}_{x} dV- {\delta c}_{y}\cdot {\int }_{{\Omega }_{0}}{u}_{y} dV- {c}_{y}\cdot {\int }_{{\Omega }_{0}}\delta {u}_{y} dV- \delta {c}_{z}\cdot {\int }_{{\Omega }_{0}}{\varvec{z}}\times {\varvec{u}} dV-{c}_{x}\cdot {\int }_{{\Omega }_{0}}\delta {u}_{x} dV- {c}_{z}\cdot {\int }_{{\Omega }_{0}}{\varvec{z}}\times \delta {\varvec{u}} dV.$$

In Eq. (7), ${\varvec{P}}$ is the first Piola Kirchhoff stress tensor, ${\varvec{F}}$ is the deformation gradient tensor,$\delta {\varvec{u}}$,$\delta p$, ${\delta P}_{LV,\mathrm{cav}}$, ${\delta c}_{x}$, ${\delta c}_{y}, {\delta c}_{z}$ are the variation of the displacement field, Lagrange multipliers for enforcing incompressibility and volume constraint, zero mean translation along *x* and *y* directions and zero mean rotation along *z* direction, respectively. The Euler–Lagrange problem then becomes finding ${\varvec{u}}{\in H}^{1}\left({\Omega }_{0}\right), p\in {L}^{2}\left({\Omega }_{0}\right),{P}_{LV,\mathrm{cav}}\in {\mathbb{R}},{c}_{x}\in {\mathbb{R}} ,{c}_{y}\in {\mathbb{R}}, {c}_{z}\in {\mathbb{R}}$ that satisfies,

8 $$\delta \mathcal{L}\left({\varvec{u}},p,{P}_{LV},{c}_{x},{c}_{y},{c}_{z}\right)=0 ,$$

and the Dirichlet boundary condition ${\left.{\varvec{u}}.{\varvec{n}}\right|}_{base}=0$ that constrains basal deformation to be in-plane as in a previous study^38^ $\forall \delta {\varvec{u}}{\in H}^{1}\left({\Omega }_{0}\right), \delta p\in {L}^{2}\left({\Omega }_{0}\right),{\delta P}_{LV}\in {\mathbb{R}},{\delta c}_{x}\in {\mathbb{R}},\delta {c}_{y}\in {\mathbb{R}},\delta {c}_{z}\in {\mathbb{R}}.$

The displacement ${\varvec{u}}$ field and the Lagrange multiplier $p$ (for enforcing local incompressibility) are discretized using quadratic and linear triangular elements. The nonlinear system of equations is linearized and solved using the Newton method using the FEniCS library^49^. An explicit time integration with a fixed time step of 1 ms is used for solving the ODEs given in Eqs. (1a–1e) and the simulations are terminated once steady-periodic state is reached.

### Incorporation of myofiber disarray

Myofiber disarray is incorporated through a structure tensor ${\varvec{H}}$^50^ describing a conical dispersion of myofibers about a mean myofiber direction ${\mathbf{e}}_{{f}_{0}}.$ A conical dispersion is considered here due to a lack of data related to the distribution of the orientations of the myofibers in HCM. For this reason, we consider the simplest possible type of dispersion where myofibers are transversely isotropic and distributed with rotational symmetry about a mean fiber direction. The corresponding structure tensor is given by

9 $${\varvec{H}}=\kappa {\varvec{I}}+\left(1-3\kappa \right){{\varvec{e}}}_{{{\varvec{f}}}_{0}}\otimes {{\varvec{e}}}_{{{\varvec{f}}}_{0}},$$

where ${\varvec{I}}$ is the identity tensor and $\kappa$ parameterizes the degree of anisotropy and myofiber disarray. At the lower limit of the disarray parameter ($\kappa =0)$, the myofibers are perfectly aligned along the ${{\varvec{e}}}_{{{\varvec{f}}}_{0}}$ direction (i.e., the structure tensor reduces to ${{\varvec{e}}}_{{{\varvec{f}}}_{0}}\otimes {{\varvec{e}}}_{{{\varvec{f}}}_{0}})$. At the upper limit of the disarray parameter $(\kappa =1/3)$, the structure tensor reduces to $1/3{\varvec{I}}$**,** representing a distribution of myofibers that produces an isotropic material response (i.e., a complete myofiber disarray).

### Constitutive relation

Mechanical behavior of the LV is described using an active stress formulation in which the first Piola Kirchhoff stress tensor ${\varvec{P}}$ was decomposed additively into a passive component ${{\varvec{P}}}_{\mathrm{ p}}$ and an active component ${{\varvec{P}}}_{\mathrm{ a}}$ (i.e. ${\varvec{P}}={{\varvec{P}}}_{\mathrm{a}}+{{\varvec{P}}}_{\mathrm{p}})$. The passive stress tensor is defined based on the strain energy function of the Holzapfel-Ogden constitutive model^51–53^ given as

10a $$W= \frac{a}{2b}{e}^{b({I}_{1}-3)}+ {\sum }_{i=f,s}\frac{{a}_{i}}{{2b}_{i}}{[e}^{[{b}_{i}{({I}_{4i}-1)}^{2}]}-1]+ \frac{{a}_{fs}}{2{b}_{fs}} [{e}^{\left({b}_{fs}{I}_{8fs}^{2}\right)}-1],$$

where

10b $${\varvec{C}}={{\varvec{F}}}^{{\varvec{T}}}{\varvec{F}}, {I}_{1}=tr\left({\varvec{C}}\right), {I}_{4f}= {\varvec{C}}:{\varvec{H}}, {I}_{4i}= {{\varvec{e}}}_{{\varvec{i}}0}\cdot \left({\varvec{C}}{{\varvec{e}}}_{{\varvec{i}}0}\right), {I}_{8fs}={{\varvec{e}}}_{{\varvec{f}}0}\cdot \left({\varvec{C}}{{\varvec{e}}}_{{\varvec{s}}0}\right).$$

In Eq. (10b), ${\varvec{C}}$ is the right Cauchy-Green deformation tensor, ***F*** is deformation gradient, ${\varvec{H}}$ is the structure tensor, ${I}_{1}, {I}_{4i}, {I}_{8fs}$ are invariants and ${{\varvec{e}}}_{{\varvec{i}}0}$ with *i* ∈ (*s, n*) is a unit vector in the myocardial fiber (*f*), sheet (*s*) and sheet normal (*n*) directions. The effect of myofiber disarray is incorporated via the invariant ${I}_{4f}$. Material parameters of the passive constitutive model are denoted by $a, b, {a}_{f}, {b}_{f}, {a}_{s}, {b}_{s}, {a}_{fs}$ and *b*_*fs*_ .

Active stress is calculated based on a previously developed active contraction model^40,54,55^. Specifically, the second Piola Kirchhoff stress tensor, ${{\varvec{S}}}_{{\varvec{a}}},$ is given as

11a $${{\varvec{S}}}_{{\varvec{a}}}=\boldsymbol{ }{T}_{max}\left(\frac{{Ca}_{0}^{2}}{{Ca}_{0}^{2}+ {ECa}_{50}^{2}}\right){C}_{t}{\varvec{H}},$$

where ${\varvec{H}}$ is the structure tensor, *T*_*max*_ is a scaling parameter that characterizes the myofiber contractility and ${Ca}_{0}$ denotes the peak intracellular calcium concentration. We note that when $\kappa =0$ (perfect alignment of the myofiber), ${\varvec{H}}={{\varvec{e}}}_{{{\varvec{f}}}_{0}}\otimes {{\varvec{e}}}_{{{\varvec{f}}}_{0}}$ and the active stress is directed only in the mean myofiber direction ${{\varvec{e}}}_{{{\varvec{f}}}_{0}}.$ The first Piola Kirchhoff active stress tensor is given as ${{\varvec{P}}}_{{\varvec{a}}}=\boldsymbol{ }{\varvec{F}}{{\varvec{S}}}_{{\varvec{a}}}.$ Length dependent calcium sensitivity ${ECa}_{50}$ and the variable *C*_*t*_ are given by

11b $${ECa}_{50}=\frac{{\left({Ca}_{0}\right)}_{max}}{\sqrt{\mathrm{exp}\left(B\left(l- {l}_{0}\right)\right)-1}} ,$$

11c $${C_t} = \left\{ {\begin{array}{ll} {\frac{1}{2}\left( {1 - \cos \left( {\frac{{\pi t}}{{{t_0}}}} \right)} \right);}&\quad {0 \le t < {t_r}~}\\ {\frac{1}{2}\left( {1 - \cos \left( {\frac{{\pi {t_r}}}{{{t_0}}}} \right)} \right){e^{ - \frac{{(t - {t_r})}}{\tau } - }};}&\quad {t \ge {t_r}.} \end{array}} \right.$$

In Eq. (11b), *B* is a constant, ${\left({Ca}_{0}\right)}_{max}$ is the maximum peak intracellular calcium concentration and ${l}_{0}$ is the sarcomere length at which no active tension develops. In Eq. (11c), ${t}_{0}$*, *${t}_{r}$ and $\tau$ are the time taken to reach peak tension, the duration of relaxation and the relaxation time constant, respectively. The sarcomere length $l$ is calculated from the myofiber stretch ${\lambda }_{\mathrm{LV}}$ by

11d $${\lambda }_{LV}=\sqrt{tr\left({\varvec{H}}{\varvec{C}}\right)} ,$$

11e $$l={\lambda }_{LV} {l}_{r} ,$$

where ${l}_{r}$ is the relaxed sarcomere length.

### Simulation cases and protocol

For each subject-specific LV FE model, the following simulations were performed sequentially.

**Figure 2:**
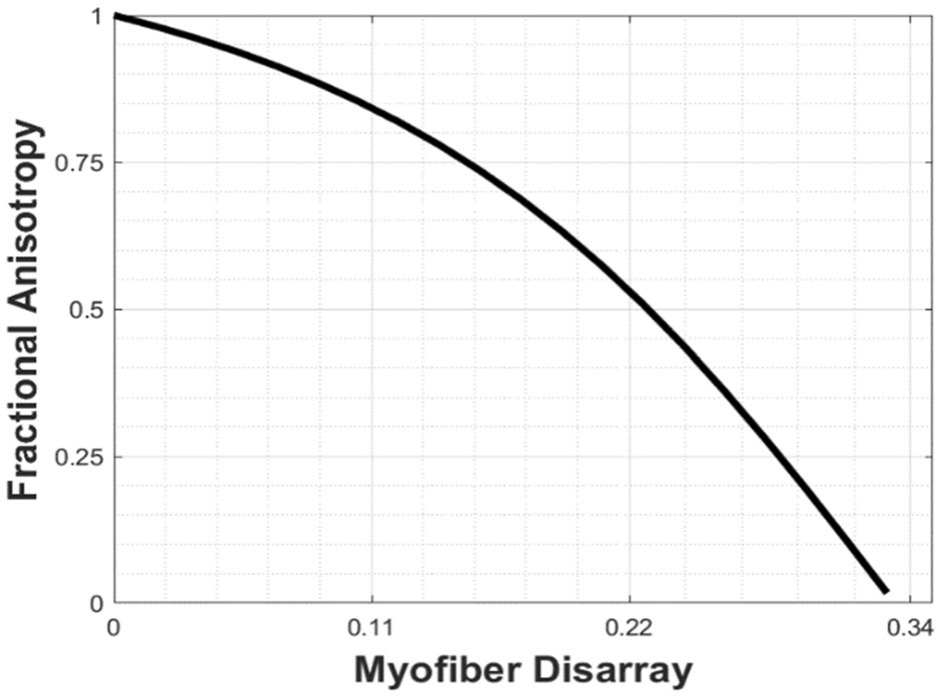
Relationship between fractional anisotropy and myofiber disarray.

1. *Estimating the unloaded geometry*: First, the unloaded LV configuration was estimated from the LV geometry reconstructed from the MR images at ED using a backward displacement method^56^. To do so, passive material parameters in the Holzapfel-Ogden model were calibrated manually so that EDPVR of the LV FE model matches that derived from the single-beat estimation by Klotz et al.^57,58^, which was also applied for HCM subjects.
2. *Simulation of a beating heart without myofiber disarray (*$\kappa$ = *0)*: Following the estimation of unloaded geometry, the unloaded LV FE model was coupled to a closed-loop lumped parameter model of the circulatory system to predict cardiac hemodynamics and mechanics. Myofiber contractility parameter *T*_*max*_ in the active contraction model, resistances and compliances in the circulatory model in each subject-specific model were calibrated without myofiber disarray (i.e., $\kappa$ = 0) to match the corresponding measured volume waveforms, blood pressure and peak GLS. Obstruction of the LVOT (due to asymmetrical septal hypertrophy and systolic anterior motion of the mitral valve that produce a large resistance to flow during the ejection phase^59^) was simulated in the obstructive HCM patient case by increasing the aortic valve resistance ${R}_{ao}$. The aortic valve resistance was increased to produce a large difference of about 60 mmHg between the peak LV pressure and the corresponding measured blood pressure, which agrees with previous clinical studies^21,22^.

3. *Simulation of a beating heart with disarray (*$\kappa$ > *0)*: Thereafter, the relationship between myofiber disarray (i.e., $\kappa$ in Eq. 9) and myofiber contractility *T*_*max*_ was investigated in the 2 HCM patients. To do so, different values of $\kappa$ were imposed globally into the HCM LV FE models based on fractional anisotropy (FA) measured in HCM patients in previous studies^31–61^. The relationship between FA and myofiber disarray (Fig. 2) was established by assuming the structure tensor ${\varvec{H}}$ is equivalent to the diffusion tensor measured in the diffusion-tensor MR images (DTMRI). Following the formulation described in Mukherjee et al.^62^, the eigenvalues (${\lambda }_{1}, {\lambda }_{2}, {\lambda }_{3}$) of the structure tensor were used to compute the FA based on the following relationship:
  12 $$FA = ~\frac{{\sqrt {{{\left( {{\lambda _1} - {\lambda _2}} \right)}^2} + ~{{\left( {{\lambda _2} - {\lambda _3}} \right)}^2} + ~{{\left( {{\lambda _3} - {\lambda _1}} \right)}^2}} }}{{\sqrt {2\left( {\lambda _1^2 + ~\lambda _2^2 + ~\lambda _3^2} \right)} }}.$$

Based on the reported FA, the range of myofiber disarray parameter $\kappa$ considered here lies between 0.0 and 0.22. For each value of $\kappa ,$ myofiber contractility *T*_*max*_ in the active contraction model was adjusted to match the clinical data. We note that the venous resting volume was also adjusted in the obstructive HCM subject in order to keep the EDV at the same value as the measurement and to maintain a pressure gradient across the LVOT as prescribed in previous studies.

### Postprocessing of simulation

The following quantities were obtained for each simulation of the 3 subjects. Specifically, total normal stress of the myofibers was determined by

13 $${S}_{f}={{\varvec{S}}:{\varvec{H}}},$$

where ${{\varvec{S}}}$ is the second Piola-Kirchoff stress and ***H*** is the structure tensor. respectively. Normal Green–Lagrange strain ${E}_{f}$ of the myofibers was determined by

14a $${E}_{f}=\boldsymbol{ }{\varvec{E}}:{\varvec{H}},$$

14b $${{\varvec{E}}}=({\varvec{C}}-{\varvec{I}})/2.$$

We note that in the limiting case $\kappa$ = 0 (perfect alignment of myofibers), ${E}_{f}={{\varvec{e}}}_{{{\varvec{f}}}_{0}}\cdot \boldsymbol{ }{\varvec{E}}\cdot {{\varvec{e}}}_{{{\varvec{f}}}_{0}}$ and ${S}_{f}={{\varvec{e}}}_{{{\varvec{f}}}_{0}}\cdot \boldsymbol{ }{\varvec{S}}\cdot {{\varvec{e}}}_{{{\varvec{f}}}_{0}}$. These stress and strain quantities were used to compute the work density of the myofiber over a cardiac cycle by

15 $${W}_{f}=\boldsymbol{ }{\int }_{Cardiac cycle}{S}_{f }d{E}_{f}.$$

Global longitudinal strain was calculated from the right Cauchy-Green stretch tensor with end diastole as the reference configuration ${{\varvec{C}}}_{{\varvec{E}}{\varvec{D}}}$ by^39^

16 $${e}= \left(1-\frac{1}{{{\varvec{e}}}_{{\varvec{l}}}\cdot {{\varvec{C}}}_{{\varvec{E}}{\varvec{D}}}\cdot {{\varvec{e}}}_{{\varvec{l}}}}\right)/2.$$

### Determination of difference between model prediction and measurements

Relative difference between the model predicted EDPVR and the one based on the empirical relationship by Klotz et al.^57,58^ is defined as

17 $${err}_{passive}= \sum_{i=1}^{N}{({P}_{klotz}({V}_{i}) -{P}_{model}({V}_{i}))}^{2}/\sum_{i=1}^{N}{({P}_{model}({V}_{i}))}^{2},$$

where ${P}_{klotz}\left(V\right)$ and ${P}_{model}(V)$ are the pressures at the same volume $V$ and $N$ is the number of equally-distributed volume data points in the EDPVR for calculation of the difference. On the other hand, the relative difference between the model predicted and clinical measurements of pressure and volume waveforms over a cardiac cycle is defined as

18 $${err}_{cardiaccycle}= \sum_{i=1}^{M}{({y}_{clinical}({t}_{i}) -{y}_{model}({t}_{i}))}^{2}/\sum_{i=1}^{M}({{y}_{model}({t}_{i}))}^{2}.$$

In Eq. (18), ${y}_{clinical}$ $\epsilon \{{P}_{clinical}, {V}_{clinical}\}$ and ${y}_{model}$ $\epsilon \{{P}_{model}, {V}_{model}\}$ are, respectively, clinical measurements and model predictions of LV pressure and volume at a particular time point $t$ in the cardiac cycle. Also, $M$ is the number of equally-distributed time steps over a cardiac cycle used to calculate the difference. Relative difference between clinical measurements and model prediction of peak GLS and blood pressure was also calculated for each subject. The computational model was implemented using the open source FE library FEniCS^49^ and the code is given in the github repository (https://github.com/MJ0706/HCM-project.git).

## Results

### Clinical data

End diastolic volume (EDV) was higher in both HCM subjects (Non-obstructive: $82 \mathrm{ml}; \mathrm{Obstructive}: 115 \mathrm{ml}$) compared to the control subject ($63.13 \mathrm{ml}$). Ejection fraction was the highest in the non-obstructive HCM subject $\left(85\%\right),$ and was comparable between obstructive HCM subject ($67\%$) and the control subject ($70\%$). Absolute peak GLS was reduced substantially in the obstructive HCM subject ($13\%)$, but was comparable between the obstructive HCM subject ($19\%)$ and the control subject ($20\%$).

### LV geometry

**Figure 3:**
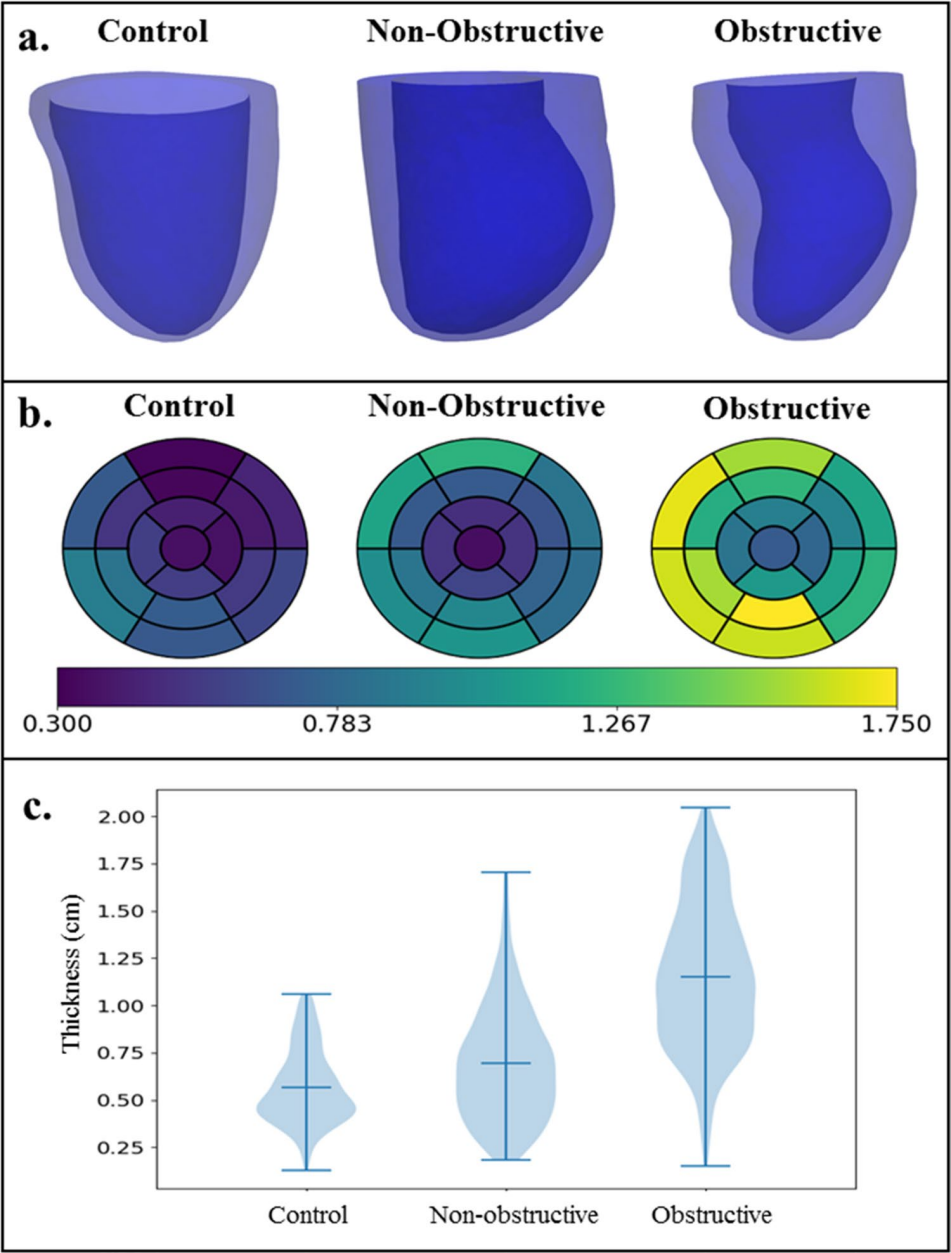
(**a**) LV geometry of the 3 subjects. (**b**) Regional distribution of wall thickness (in cm) based on AHA segmentation and (**c**) Violin plot of the wall thickness.

Left ventricular geometries reconstructed from the MR images and the regional wall thickness based on AHA segmentation for each subject are shown, respectively, in Fig. 3a and b. Septum wall thickness of the obstructive HCM subject ($1.43\pm 0.36$ cm) was the largest followed by that of the non-obstructive HCM subject ($0.85\pm 0.24$ cm) and the control subject ($0.73\pm 0.14$ cm). In each HCM subject, LV free wall thickness was smaller (cf. septum) but was larger when compared to the same region in the control subject (Obstructive HCM: $1.07\pm 0.18$ cm; Non-Obstructive HCM: $0.73\pm 0.13$ cm; Control: $0.5\pm 0.08$ cm). In Fig. 3c, the violin plot of wall thickness shows the summary statistics of regional thickness and its variation. Mean global wall thickness was higher in the HCM subjects compared to the control (Obstructive HCM: 1.27 ± 0.33 cm; Non-Obstructive HCM: 0.79 ± 0.23 cm; Control: 0.58 ± 0.15 cm). The variation between minimum and maximum wall thickness (represented by lower and upper horizontal lines) was highest in the obstructive HCM subject followed by the non-obstructive and the control subject.

### LV mechanics without consideration of myofiber disarray

**Figure 4:**
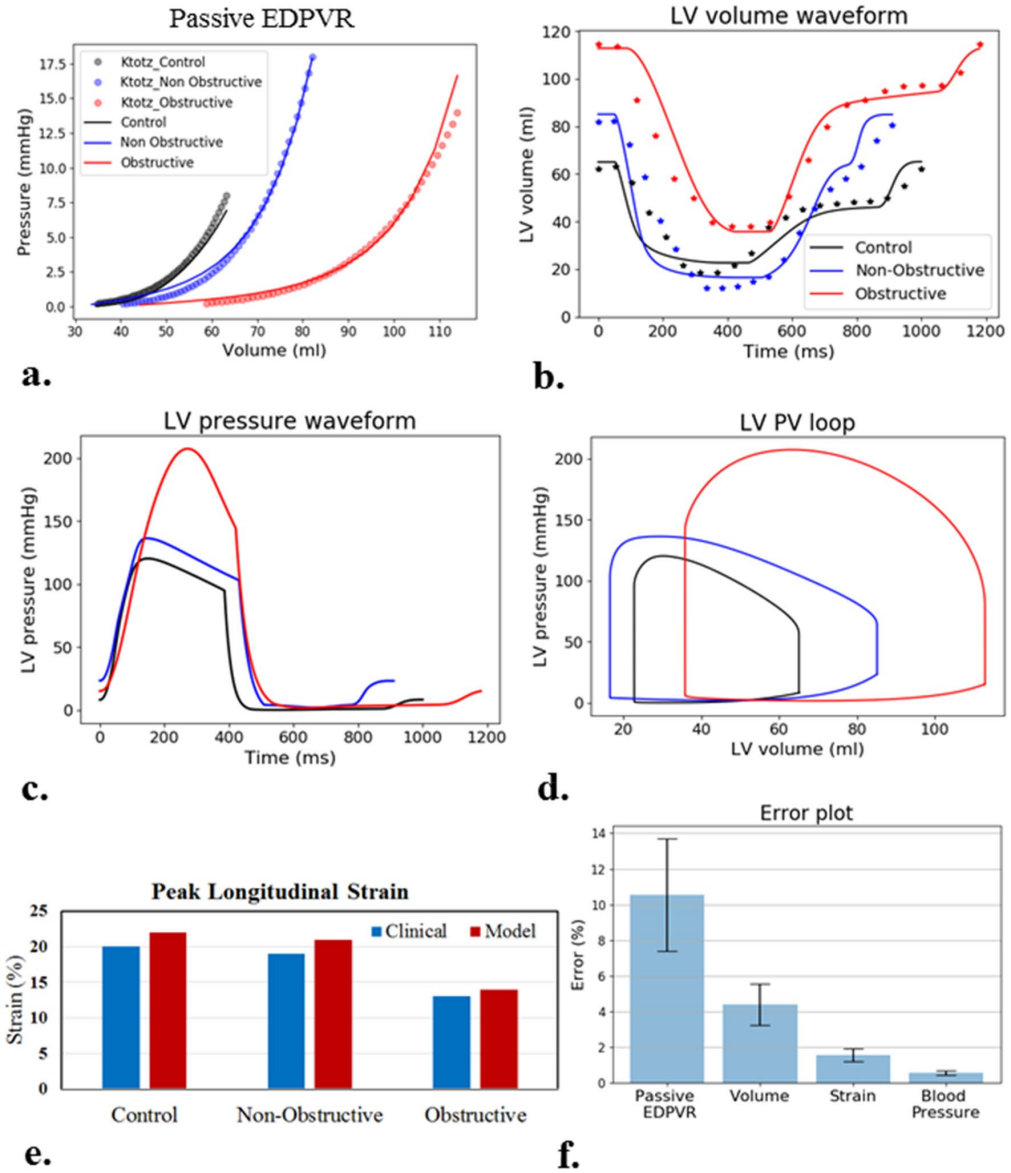
Calibration of model parameters for each subject without myofiber disarray. (**a**) EDPVR. (**b**) Volume waveforms (solid line—simulated results, dotted line—clinical results). (**c**) Pressure waveforms. (**d**) PV loop. (**e**) Peak GLS. (**f**) Difference between model predictions and measurements.

The calibrated models’ prediction of the EDPVR is consistent with that obtained from the single-beat estimation based on the Klotz relationship (Fig. 4a). The passive material properties (Appendix B.1) reflected an increased isotropic stiffness (Obstructive: 334.8%, Non-obstructive: 769.6%) and a decrease in stiffness along the fiber direction (over 99%) in both HCM patients when compared to control. The calibrated models’ predictions of LV volume waveform, blood pressure and peak GLS also agree with the corresponding patient-specific clinical measurements (Fig. 4b–e). Differences between the measurements and the model predictions are within about 10%, with the highest difference occurring in the comparison between the EDPVR derived from the empirical Klotz relationship and the model (Fig. 4f).

**Figure 5:**
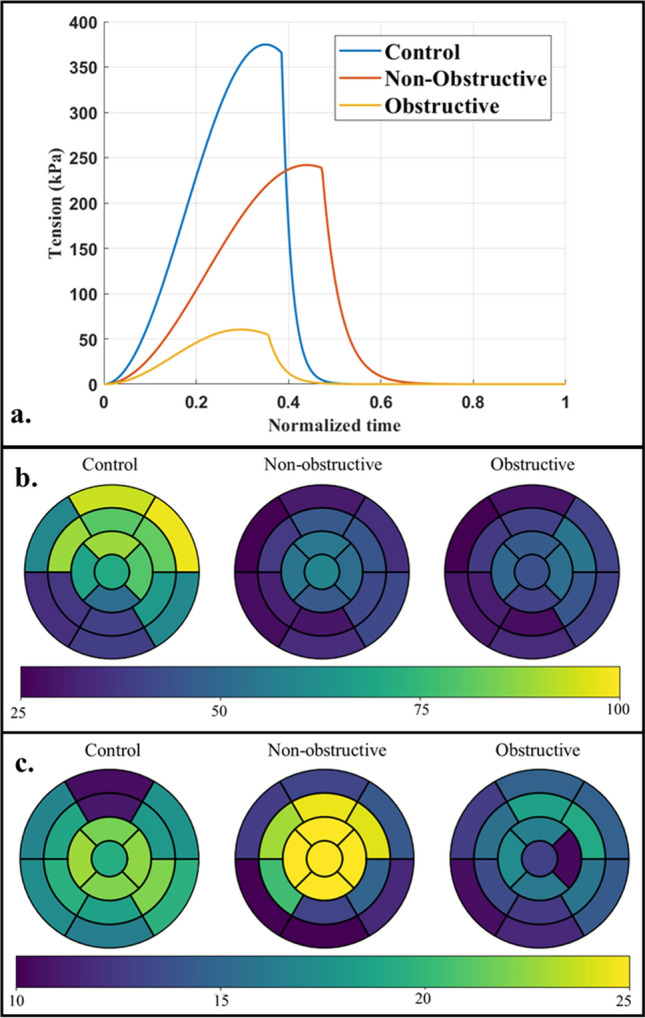
(**a**) Isometric tension plot over time normalized by cardiac cycle; regional distribution of (**b**) peak total fiber stress (in kPa) and (**c**) peak longitudinal strain (absolute value in %) for each subject.

Peak (isometric) myofiber tension derived from the calibrated active stress model parameters was substantially smaller in the HCM subjects when compared to the control subject (Fig. 5a). The obstructive HCM subject has the smallest peak myofiber tension of 60 kPa and the non-obstructive HCM subject has a peak myofiber tension of 242 kPa, which are both lower compared to that of the control subject (375 kPa). Peak myofiber stress averaged over the entire LV was the smallest in the obstructive HCM subject ($39\pm 8.85$ kPa) followed by the non-obstructive HCM subject ($40.6\pm 10.3$ kPa) and the control subject ($66.9\pm 21$ kPa) (Fig. 5b). Peak myofiber stress was lower at the septum than the LVFW in both HCM subjects, with the lowest value found in the obstructive HCM subject. Peak GLS was lower in the entire LV of the obstructive HCM subject compared to the other 2 subjects (Fig. 5c). Longitudinal strain was higher at the LVFW ($- 19.8\%$ ) compared to the septum ($-12.5\%$) in the obstructive HCM subject. In the other 2 subjects, however, the difference between longitudinal strain at the septum and LVFW was not prominent (Control: septum $-19.5\%$ vs. LVFW $-18.8\%$; non-obstructive HCM: septum $- 21.8\%$ vs LVFW $- 18.7\%$).

**Figure 6:**
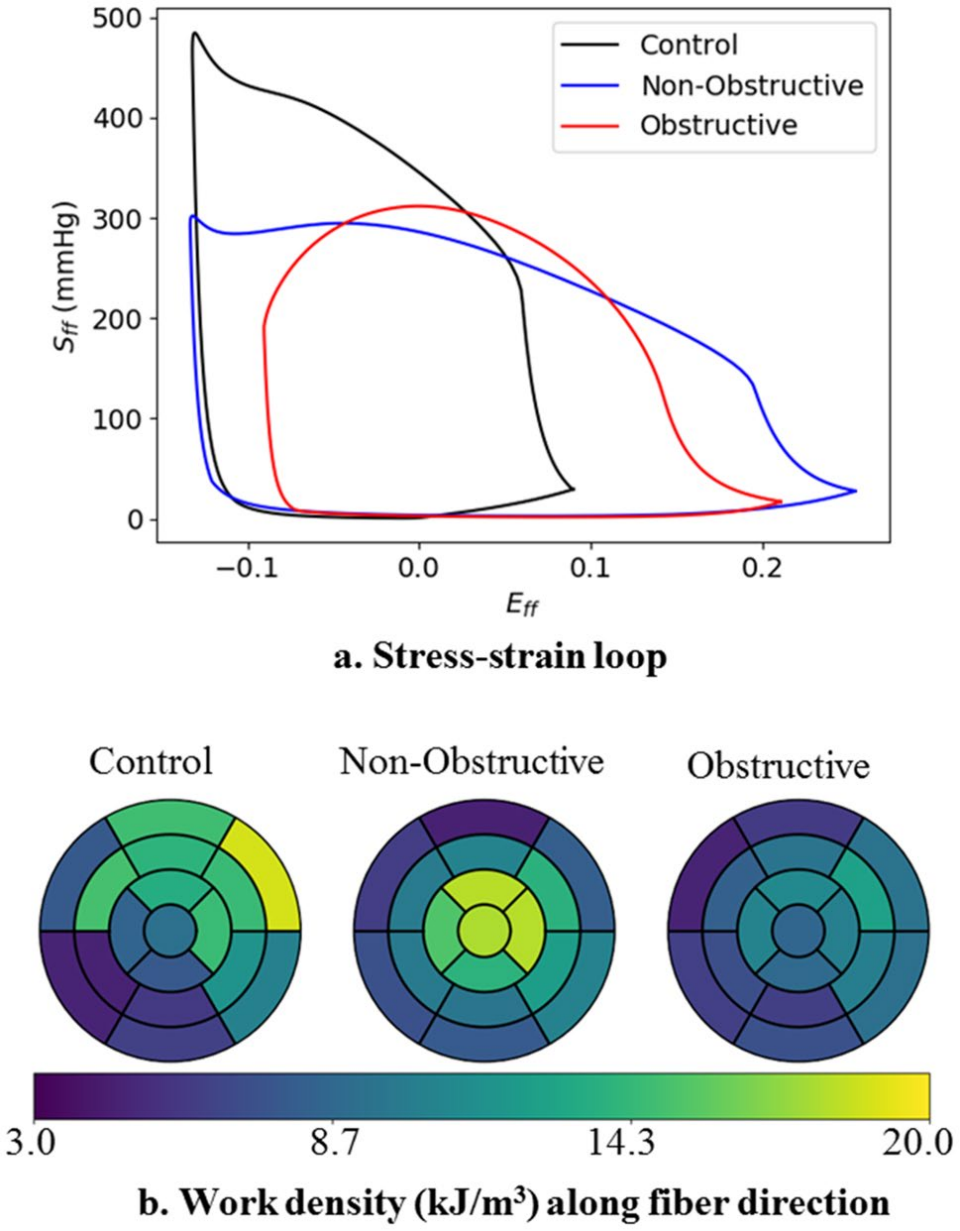
Work densities in the HCM and control subjects without myofiber disarray. (**a**) Stress–strain loop along mean fiber direction, (**b**) regional distribution of myofiber work density (in $kJ/{m}^{3}$ ).

Total myofiber work density (indexed by the area of the stress–strain loop along material direction) was the lowest in the obstructive HCM subject ($9.0\,\, \mathrm{kJ}/{\mathrm{m}}^{3}$), followed by the control subject ($11.2\,\, \mathrm{kJ}/{\mathrm{m}}^{3}$) and the non-obstructive HCM subject ($11.9 \,\,\mathrm{kJ}/{\mathrm{m}}^{3}$) (Fig. 6). In terms of its regional distribution, myofiber work density was higher at the LVFW (control: $14.2\,\, \mathrm{kJ}/{\mathrm{m}}^{3}$; non-obstructive: $13.1\,\, \mathrm{kJ}/{\mathrm{m}}^{3}$; obstructive: $10.8\,\, \mathrm{kJ}/{\mathrm{m}}^{3}$) compared to the septum (control: $8.5\,\, \mathrm{kJ}/{\mathrm{m}}^{3}$; non-obstructive: $10.1 \,\,\mathrm{ kJ}/{\mathrm{m}}^{3}$; obstructive: $7.8\,\, \mathrm{kJ}/{\mathrm{m}}^{3}$) for all subjects.

### Effects of myofiber disarray on the LV mechanics of HCM subjects

**Figure 7:**
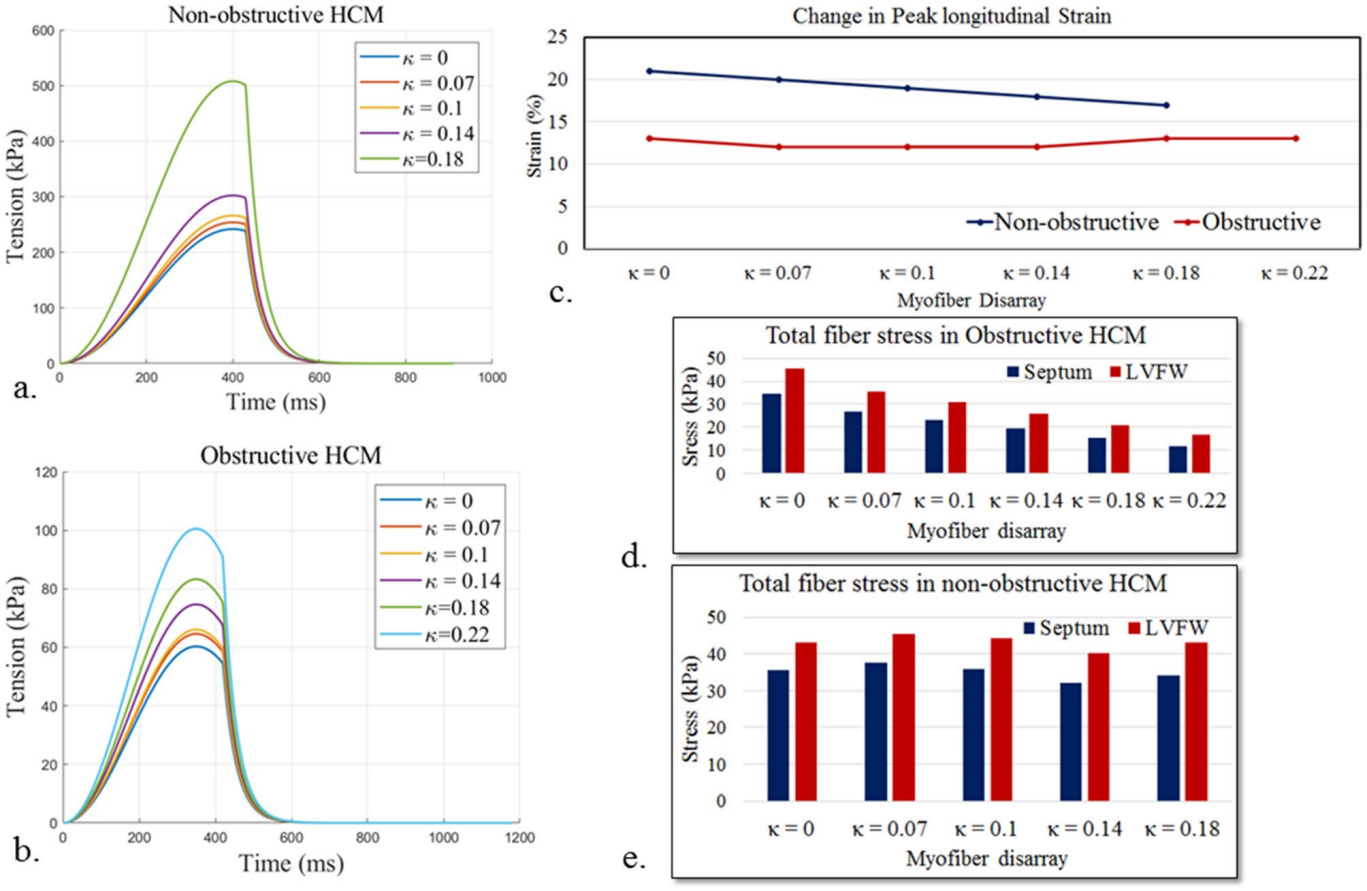
Effects of myofiber disarray. Isometric tension-time plot of (**a**) non-obstructive and (**b**) obstructive HCM subjects. (**c**) Peak GLS for the 2 HCM subjects. Peak stress of the myofibers at the septum and LVFW for (**d**) obstructive and (**e**) non**-**obstructive HCM subjects.

With an increase in myofiber disarray, it was necessary to increase the scaling parameter *T*_*max*_ (associated with myofiber contractility) to match the clinical data of the HCM subjects (see Appendix A). The resultant peak myofiber tension was therefore increased as a result with increasing myofiber disarray (Fig. 7). Specifically, peak myofiber tension associated with the largest degree of disarray was $507.9\,\,\mathrm{kPa}$ $(\kappa =0.18)$ and $100.5\,\, \mathrm{kPa}$ $(\kappa =0.22)$ for the non-obstructive and obstructive HCM patients, respectively. Peak GLS did not change substantially (~ 3%) with increasing myofiber disarray in both HCM subjects. Regional distribution of peak longitudinal strain, peak stress of the myofibers also did not change with different degree of myofiber disarray. In the obstructive HCM subject, peak stress of the myofibers was decreased in both LVFW and septum with increasing myofiber disarray (Fig. 7d). Conversely in the non-obstructive HCM subject, peak stress of the myofibers was slightly increased with increasing myofiber disarray (Fig. 7e).

**Figure 8:**
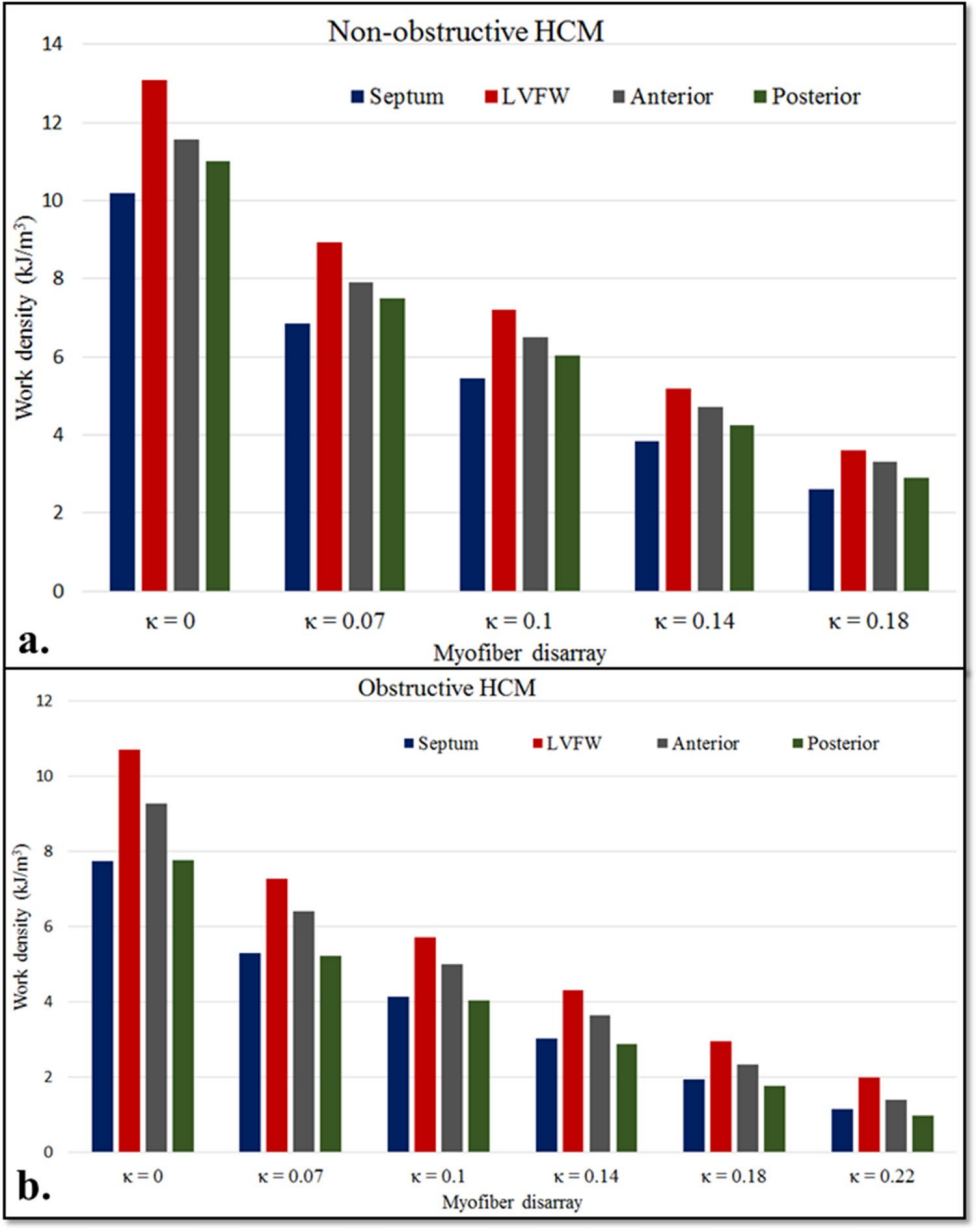
Effects of myofiber disarray on myofiber work densities for (**a**) the non-obstructive and (**b**) the obstructive HCM subject.

Myofiber work density was reduced with increasing myofiber disarray in both non-obstructive and obstructive HCM subjects (Fig. 8a, b). The reduction in myofiber work density was the highest in the septum and lowest in the anterior in the non-obstructive HCM subject (Septum: − 74%; Anterior: − 71% at $\kappa =0.18$ cf. $\kappa =0.0)$. On the other hand, in obstructive HCM subject, posterior and LVFW regions have the highest and lowest reduction in myofiber work density, respectively (Posterior: − 87%; LVFW: − 81% at $\kappa =0.22$ cf. $\kappa =0.0)$.

## Discussion

We have developed a patient-specific computational framework of the LV mechanics to investigate the effects of myofiber disarray using clinical data from 2 HCM subjects with different phenotypes (obstructive vs non-obstructive) along with a control subject. The key finding of this study suggests that the contractile force generated by the cardiac muscle cell is smaller in the obstructive HCM subject compared to the control subject. In the non-obstructive HCM subject, the contractile force is smaller only if the degree of global myofiber disarray $\kappa$ is less than 0.14. Specifically, the study found that the contractile force generated by the cell to reproduce the clinical measurements is larger with an increase in global myofiber disarray. An increase in myofiber disarray led to a reduction in myofiber work density in both HCM subjects.

### LV wall thickness

The reconstructed LV geometries of the HCM subjects are consistent with those reported in previous clinical studies. Specifically, the maximum LV wall thickness in the obstructive and non-obstructive HCM subjects are 17.4 mm and 12.3 mm at the mid posterior and basal anterior wall, respectively. These values are consistent with previous studies^63,64^. The ratio of maximum septum wall thickness to minimum posterior wall thickness for the non-obstructive (1.9) and obstructive (1.54) HCM subjects are also within the threshold (≥ 1.3) used to define asymmetric septal hypertrophy in HCM patients^65^.

### LV function

Both HCM subjects have higher EDV than the control subject (Fig. 3), with the highest value found in the obstructive HCM subject. Ejection fraction is normal (67%) and supra-normal (85%) for the obstructive and non-obstructive HCM subjects, respectively. The supra-normal EF in the non-obstructive HCM patient is a result of its small ESV. Peak GLS is slightly smaller in the non-obstructive HCM subject (19%) compared to the control subject (20%), but is substantially smaller in the obstructive HCM subject (13%). The smaller peak GLS in the obstructive HCM subject is within the range of − 9.65% to − 16% reported in previous studies^23,66^. It has been suggested in some studies that peak GLS is a sensitive indicator of global LV function as well as a prognostic marker to predict mortality and cardiac events in HCM and heart failure with preserved ejection fraction (HFpEF)^67–70^. The mechanism by which GLS is associated to LV function and mortality is, however, not quite clear.

The results suggest that the reduction in peak GLS is associated with a reduction in myofiber contractility that is indexed by the peak muscle fiber tension. Without considering myofiber disarray, the models predicted that the peak tension to reproduce the clinical measurements is, respectively, 84% (absolute) and 35% (absolute) smaller in the obstructive and non-obstructive HCM subject when compared to the control subject. By considering myofiber disarray based on the range found in DTMRI studies with $\kappa$ having values between 0 to $0.22$, we found that the peak muscle fiber tension has to increase to compensate for an increasing degree of myofiber disarray in order to reproduce the clinical measurements. Within this range of $\kappa$, peak GLS varies by only ± 2% (absolute) in the obstructive HCM subject and is still depressed compared to the control subject (Fig. 7c). At the highest degree of myofiber disarray in the obstructive HCM subject, however, the peak muscle fiber tension is still about 73% (absolute) lower than that in the control subject. For the non-obstructive HCM subject, we found that the peak muscle fiber tension is equivalent to the control subject at a disarray $\kappa$ = 0.14. At that value of $\kappa$, peak GLS is − 18% and lies within the ranges reported previously^71,72^. These findings therefore suggest the myocardial contractile stress generated in vivo is likely reduced in the HCM subjects, especially in the obstructive phenotype, which can explain the results of a previous MRI study on HCM patients showing that a reduction in FA is associated with a reduction in myocardial strain^60^. The reduction in myocardial contractile stress in the HCM subjects is associated with the increase in LV wall thickness.

The finding that a reduced peak GLS is associated with a reduction in myocardial contractility even with a normal EF is consistent with a previous modeling study based an idealized LV geometry^38^. In that study, only a reduction in myocardial contractility can explain the simultaneous features (including a reduction in GLS) found in patients with HFpEF. Specific to HCM, a reduction in myocardial contractility has also been found in animal studies and is attributed to the mutation of sarcomeric protein^73,74^. The lower peak tension found here is also consistent with the reduced myofibril density found in vitro studies of myocytes obtained from myocardial biopsies of HCM patients^28^.

### Myofiber stress

Peak stress of the myofibers is heterogeneously distributed in the LV (Fig. 5b). Compared to the control subject, peak myofiber stress is smaller in the HCM subjects, and is smallest in the obstructive HCM subject. This result is largely due to the increase in wall thickness in the HCM subject, and is consistent with previous studies of HCM patients^35,75^. Peak myofiber wall stress is also lower in the septum (thicker region) than LVFW (thinner region) in all subjects. Between non-obstructive and obstructive HCM subjects, peak stress of the myofibers behaves differently with increasing myofiber disarray (Fig. 5d, e). With an increase in myofiber disarray, peak stress of the myofibers increases in the non-obstructive HCM subject, but decreases in obstructive HCM subject. This result suggests that the effects of myofiber disarray on myofiber stress may be sensitive to geometry.

### Distribution of work

Global myocardial work density, indexed by the pressure volume area, is linearly correlated to the cardiac metabolism and total myocardial oxygen consumption^76–78^. Local myofiber work density ${W}_{f}$ is determined from the area in the average myofiber stress–strain loop (Fig. 6). Without consideration of myofiber disarray, our analysis shows that the non-obstructive HCM subject has the highest mean ${W}_{f}$ ($11.9\,\, \mathrm{kJ}/{\mathrm{m}}^{3}$), followed by the control subject ($11.2\,\, \mathrm{kJ}/{\mathrm{m}}^{3}$), and the obstructive HCM subject ($9.00\,\, \mathrm{kJ}/{\mathrm{m}}^{3}$). With disarray where cardiac muscles are oriented in other directions other than the mean myofiber direction, ${W}_{f}$ decreases with increasing degree of myofiber disarray in both HCM subjects (Fig. 8). These results showing a lower ${W}_{f}$ in the obstructive HCM subject than the non-obstructive HCM subject (and the normal) is consistent with published results of myocardial work index (pressure-strain loop area) assessed noninvasively using echocardiography and blood pressure measurement in HCM patients^79,80^. The findings that septal ${W}_{f}$ is lower than that in the LVFW are also consistent with these studies, especially when in HCM phenotypes with substantial septal hypertrophy. We note that ${W}_{f}$ is defined differently from the myocardial work index measured in the clinic as the latter relies on a global index of stress (i.e., pressure) rather than the local stress of the myofibers. Nevertheless, both of these indices are metric of the total work of the myofiber over a cardiac cycle. Our finding suggests that the development of myofiber disarray further worsens the already lower myofiber work in the HCM subjects, further suggesting that this feature is a contributor to the lower myocardial work index found clinically in HCM patients. The lower work arises because myofibers are disoriented and not contributing efficiently to the overall contraction of the heart (e.g., myofibers oriented in the radial directions are not performing work when the wall thickens during contraction). Therefore, myofiber disarray is one of the key contributors to the worsening of myocardial work in HCM patients (in addition to other features such as mechanical dyssynchrony).

## Limitation

There are some limitations associated with this study. First, this is a pilot study using only 2 HCM subjects with 2 broadly different phenotypes (i.e., obstructive and non-obstructive HCM). As such, the results can only provide some indications on the mechanics as well as the relationship between myofiber disarray on myofiber contractility in HCM. Nevertheless, the study can be extended in future to consider the broad range of disease pattern and variation of morphological phenotypes (such as apical hypertrophy) found in HCM patients. Second, we assumed a conical dispersion of the myofiber along a mean direction and did not consider diffused and regional myofiber disarray that may be present in HCM patients. Because previous studies have suggested that physiological transmural variation of the helix angle associated with the perfectly aligned muscle fibers is optimal in terms of generating the largest stroke volume^81^, it implies that any deviation from the physiological arrangement of muscle fibers (e.g., myofiber disarray) will be sub-optimal. As such, we do not expect the type of dispersion to significant affect our findings that the contractile force generated by the tissue to produce the same LV function is increased with an increase in myofiber disarray (i.e., myocardium becomes less efficient) (see Fig. 7a and b). Local DTMRI measurements of myofiber disarray in HCM patients, however, can be incorporated to develop more personalized model of HCM in future studies. Third, we did not consider the presence of local or diffuse fibrosis in HCM. Patient-specific cardiac magnetic resonance imaging with late gadolinium enhancement can be used to quantify local fibrosis, that in turn, can be applied into the model. Fourth, we assumed homogeneous contraction and did not consider electrophysiology in the model because the key goal here is to investigate the isolated effects of myofiber disarray and geometry on ventricular mechanics in HCM patients. Nevertheless, arrythmia and mechanical dyssynchrony can be present in HCM patients^82^ and previous computer models^45,83^ have shown that mechanical dyssynchrony (without myofiber disarray and geometrical remodeling) worsens the cardiac function (i.e., reduce LV pressure and stroke volume). Correspondingly, we expect the presence of mechanical dyssynchrony to exacerbate the adverse effects of myofiber disarray and ventricular geometrical remodeling in HCM as found here. Future studies can apply computer models coupling cardiac electrophysiology and mechanics^44,45,84,85^ to investigate the combined effects of mechanical dyssynchrony, geometrical remodeling and myofiber disarray. Fifth, beside asymmetric hypertrophy of the LV wall, HCM is also associated with abnormal mitral valve morphology and function (i.e., systolic anterior motion, SAM) that can contribute to obstruction of LVOT and mitral valve regurgitation^83–85^. We did not consider mitral valve regurgitation (MVR) in our model because we did not see any substantial systolic anterior motion of the mitral valve leaflets causing regurgitation. Nevertheless, future studies can consider the effects of abnormal MV morphology on global hemodynamics in HCM patients by incorporating the mitral valve along with the papillary muscles and chordae tendineae^86^. Last, we did not consider the effects of myofiber disarray and an increase thickness of the septum wall on right ventricular function, which may in turn, affects the left ventricular function via interventricular interactions. This limitation can be addressed in future studies using a FE modeling framework consisting of the biventricular unit^87–90^.

## Conclusion

We have developed patient-specific computational models based on clinical data acquired in 2 HCM (obstructive and non-obstructive) patients and a control subject to investigate LV mechanics and the relationship between myofiber disarray and myofiber contractility in this disease. Using these models, we show that myofiber contractility is increased to compensate for an increase in myofiber disarray associated with HCM in order to maintain same LV function. For the range of myofiber disarray measured in HCM patients, however, we found that the myofiber contractility in the obstructive HCM subject is still smaller compared to the control subject at the highest degree of myofiber disarray. Myofiber contractility of the non-obstructive HCM subject is close to that of the control subject only when myofiber disarray is substantial with a fractional anisotropy of 0.75. An increase in myofiber disarray also led to a reduction in myofiber work in the HCM subjects. These findings suggest that myofiber contractile stress generated in HCM patients is reduced and is associated with an increase in wall thickness, and the reduction in myofiber work seen in HCM patients may be due in part to myofiber disarray.

## Supplementary Information

Supplementary Information.

## Data Availability

The code and datasets generated and/or analyzed during the current study are available in the github repository, [https://github.com/MJ0706/HCM-project.git].
