# Supplementary material for: Computational analysis of ventricular mechanics in hypertrophic cardiomyopathy patients
Source: Sci Rep. 2023 Jan 18;13:958. doi: 10.1038/s41598-023-28037-w (PMC9849405; doi:10.1038/s41598-023-28037-w)
Supplement: Supplementary file 1 — Supplementary Information. [file 41598_2023_28037_MOESM1_ESM.docx]

**Appendix:**

**A: PV Loop for Obstructive & non-obstructive HCM cohorts**


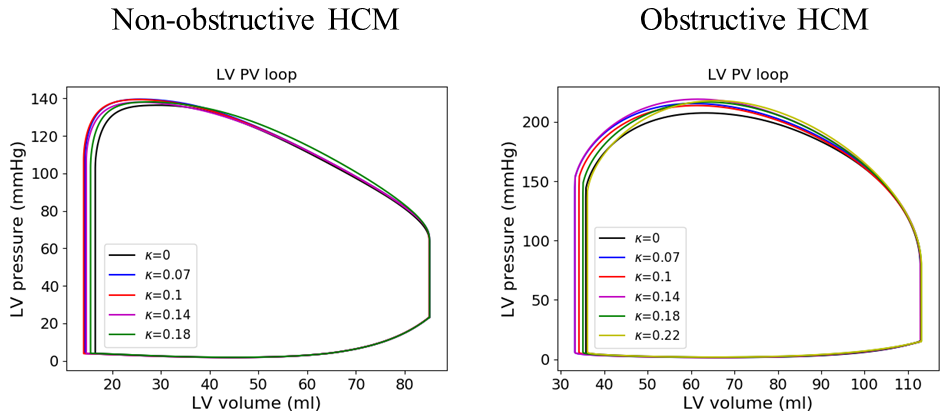


**B. Model parameters without disarray**

The calibrated passive and active material described in Eq. 10 and Eq. 11, respectively, are enlisted in Table B.1.

| **B.1: Material parameters** | | | | |
| --- | --- | --- | --- | --- |
| Parameters | Unit | Control | Non-obstructive | Obstructive |
| **Holzapfel-Ogden model** | | | | |
| a | $Pa$ | 46 | 400 | 200 |
| b |  | 12 | 5 | 4 |
| $a_{f}$ | $Pa$ | 7.51e03 | 37.5 | 15 |
| $b_{f}$ |  | 5.893 | 1.47325 | 22.1 |
| $a_{s}$ | $Pa$ | 492 | 492 | 492 |
| $b_{s}$ |  | 3.393 | 3.393 | 3.393 |
| $a_{fs}$ | $Pa$ | 70 | 70 | 70 |
| $b_{fs}$ |  | 3.929 | 3.929 | 3.929 |
| **Guccione model** | | | | |
| $T_{max}$ | $kPa$ | 620 | 400 | 99.75 |
| $\tau$ | $ms$ | 20 | 35 | 35 |
| $t_{trans}$ | $ms$ | 385 | 430 | 420 |
| B |  | 4.75 | 4.75 | 4.75 |
| $t_{0}$ | $ms$ | 350 | 400 | 350 |
| $l_{0}$ | $\mu m$ | 1.55 | 1.55 | 1.55 |
| ${Ca}_{0}$ | $\mu M$ | 4.35 | 4.35 | 4.35 |
| ${Ca}_{0max}$ | $\mu M$ | 4.35 | 4.35 | 4.35 |
| $l_{r}$ | $\mu m$ | 1.85 | 1.85 | 1.85 |
| BCL | $ms$ | 1000 | 910 | 1180 |

The model parameters prescribed in circulatory model and time varying elastance model are enlisted in Table B.2.

| **B.2: Circulatory and left atrium model parameters** | | | | |
| --- | --- | --- | --- | --- |
| Parameter | Unit | Control | Non-obstructive | Obstructive |
| **Circulatory model** | | | | |
| $C_{a,p}$ | $ml Pa$ | 0.00208 | 0.00544 | 0.0048 |
| $C_{a,d}$ | $ml Pa$ | 0.02145 | 0.0561 | 0.0495 |
| $C_{ven}$ | $ml Pa$ | 0.196 | 0.378 | 0.014 |
| $V_{a,p,0}$ | $ml$ | 144 | 144 | 306 |
| $V_{a,d,0}$ | $ml$ | 160 | 160 | 160 |
| $V_{ven,0}$ | $ml$ | 4500 | 3100 | 4525 |
| $R_{ao}$ | $Pa ms {ml}^{-1}$ | 3000 | 3000 | 31500 |
| $R_{ven}$ | $Pa ms {ml}^{-1}$ | 10 | 10 | 100 |
| $R_{a,p}$ | $Pa ms {ml}^{-1}$ | 108000 | 90000 | 45000 |
| $R_{a,d}$ | $Pa ms {ml}^{-1}$ | 127200 | 84800 | 159000 |
| $R_{mv}$ | $Pa ms {ml}^{-1}$ | 1000 | 1000 | 1000 |
| **Time varying elastance model** | | | | |
| $E_{es,la}$ | $Pa/ml$ | 9 | 7 | 10 |
| $A_{la}$ | $Pa$ | 0.801 | 0.6675 | 4.005 |
| $B_{la}$ | ${ml}^{-1}$ | 0.0152 | 0.00475 | 0.021 |
| $V_{0,la}$ | $ml$ | 10 | 10 | 10 |
| $T_{max, la}$ | $ms$ | 120 | 120 | 150 |
| $\tau_{la}$ | $ms$ | 25 | 25 | 25 |
| $t_{delay,la}$ | $ms$ | 140 | 140 | 140 |

**C. Model parameters with disarray**

The model parameters calibrated to match with clinical volume waveform and blood pressure with varying degree of disarray for 2 HCM subjects are listed below. Noted, the rest of the model parameters are same as described in Appendix B.

| Parameter | Unit | $\kappa=0.07$ | $\kappa=0.1$ | $\kappa=0.14$ | $\kappa=0.18$ | $\kappa=0.22$ |
| --- | --- | --- | --- | --- | --- | --- |
| **Obstructive HCM** | | | | | | |
| $T_{max}$ | $kPa$ | 106.8 | 109.25 | 123.5 | 137.75 | 166.25 |
| $V_{ven,0}$ | $ml$ | 4550 | 4640 | 4660 | 4660 | 4660 |
| **Non-obstructive HCM** | | | | | | |
| $T_{max}$ | $kPa$ | 420 | 440 | 500 | 840 |  |
